# Single-point Mutation of an Histidine-aspartic Domain-containing Gene involving in Chloroplast Ribosome Biogenesis Leads to White Fine Stripe Leaf in Rice

**DOI:** 10.1038/s41598-017-03327-2

**Published:** 2017-06-12

**Authors:** Changwei Ge, Li Wang, Weijun Ye, Liwen Wu, Yongtao Cui, Ping Chen, Jiangjie Pan, Dong Zhang, Jiang Hu, Dali Zeng, Guojun Dong, Qian Qian, Longbiao Guo, Dawei Xue

**Affiliations:** 1 State Key Laboratory of Rice Biology, China National Rice Research Institute, Hangzhou, 310006 China; 2 College of Life and Environmental Sciences, Hangzhou Normal University, Hangzhou, 310036 China

## Abstract

Plant leaves are a crucial organ associated closely with chloroplast development, photosynthesis rate and crop productivity. In this study, a *white fine stripe leaf 1* (*wfsl1*) mutant was isolated and characterized from the *japonica* rice Zhonghua11 (ZH11) after ethyl methanesulfonate mutagenesis. The *wfsl1* displayed white fine stripe leaves since tillering stage and abnormal chloroplast structure. Map-based cloning and Bioinformatic analysis indicated that *WFSL1* on chromosome 1 contains an “A” to “T” substitution in protein coding region, and encodes a putative metal-dependent phosphohydrolase with HD domain at the N-terminus. WFSL1 was targeted to the chloroplasts and had higher expression in mature leaves and sheaths. RNA-seq analysis revealed that chloroplast development and photosynthesis genes were significantly affected in *wfsl1* plants. Levels of WFSL1 and chloroplast encoded proteins were decreased in *wfsl1* mutants via western blot analysis. Compared with WT, *wfsl1* exhibits lower Chl content and defective in biogenesis of chloroplast ribosomes, which resulted in reduced grain yield. Taken together, our results show that WFSL1 is critical for chloroplast development, ribosome biogenesis, and light energy utilization, finally affects grain yield.

## Introduction

Chloroplast, the center for leaf cell metabolism which plays an important role in light reception and carbon sequestration in higher plants^1^. Chloroplast development is regulated by genes encoded of nuclear and plastids, but for the limited coding capacity of plastids, chloroplast development is mainly under nuclear control. The coordination of gene expression through nuclear and plastids is essential for chloroplasts biogenesis in plant^2^. The transcription of nuclear and chloroplast genes mainly depends on two RNA polymerase. One is the bacterial-type nuclear-encoded RNA polymerase (NEP). NEP transcribes plastid genes involved *RpoA*, *B*, *C* that are necessary for the development of plastidic genetic systems in the early stage of chloroplast development. The other bacterial-type plastid-encoded RNA polymerase (PEP) is response for the photosynthesis genes (such as *RbcL*, *RbcS*, *PsbA*, *RCA*) development at mature stage^3, 4^. Owing to defects in PEP activity, the formation of thylakoid membrane and photosynthesis was repressed, suggesting it is an important role of PEP in chloroplast development^5^. Proteins encoded by the plastid genome are synthesized by plastidic prokaryotic type 70 S ribosome that are composed of 30 S and 50 S subunits^6–9^. Deficiency in content of 70 S ribosome results in stunted chloroplast development. *Arabidopsis* konck-down mutant RH22 accumulated precursors 23 S rRNA that displayed virescent phenotype. RH22 affected ribosome assembly in rRNA metabolism^10^. ObgC participated in 70 S ribosome assembly. The knock down and RNAi of *ObgC* result in chlorotic phenotype in rice^8^. Thus, 70 S ribosome biogenesis is essential for chloroplast development in higher plants.

Plant variegations are characterized by the presence of white sectors and green sectors. The white sectors contain defective chloroplasts and the green sectors contain normal chloroplasts^11^. *Immutans* (*im*) and *var2* are two types variegation mutants in *Arabidopsis*. The *im* mutant is induced by a nuclear recessive gene and the extent of variegation can be modulated by light and temperature. IM protein is a plastid homologue of the mitochondrial alternative oxidase and functions as a redox component of the phytoene desaturation pathway^11^. Mutations in the VAR2 locus cause variegation due to loss of a chloroplast thylakoid membrane protein that is similar to the FtsH family of AAA protein. FtsH functions in a number of diverse membrane-associated events and VAR2 protein functions in thylakoid membrane biogenesis^12^. Both *im* and *var2* mutants provide an excellent system to understand the molecular mechanism of nuclear-plastid interactions in *Arabidopsis*. In rice, many genes were also associated with nuclear-plastid interaction. *V1* encodes a chloroplast-localized protein NUS1 regulating chloroplast RNA metabolism^13–16^. V2 encodes a plastids/mitochondria Guanylate kinase (pt/mtGK) which catalyzes GMP to GDP in guanylate biosynthesis and affects chloroplast development. It suggests that pt/mtGK is an important part for chloroplast development^17^. *RNRL1* and *RNRS1* encode the large and small subunits of rice ribonucleotide reductase, respectively. They are necessary for the DNA replication in chloroplast^18^. *OsDVR* plays certain roles in nuclear-cytoplasmic signal transduction by which nucleus directly regulates cytoplasm^19, 20^. Leaf-color mutations are a crucial for dissection of regulation mechanism of with chloroplast development, photosynthesis and key agronomic traits.

Here, we isolated a *w* *hite* *f* *ine* *s* *tripe* *l* *eaf* (*wfsl1*) mutant in rice. The phenotype of mutant not very likely with *im* and *var2* mutants, the green and white sectors longitudinal distributed on the blade of mutants. It’s interesting that mutant phenotype mainly first occurs on the newly grown leaves and sheath at tillering stage, continued to the mature stage. Our study discovers that map-based cloning identifies *WFSL1* on chromosome 1 encoding an HD domain containing gene. The WFSL1 protein is localized to chloroplast and was decreased in *wfsl1* mutant leaves. The *wfsl1* mutant was defective in biogenesis of chloroplast ribosomes, and affected photosynthesis rate and grain yield. Our results suggest that WFSL1 is critical for chloroplast development and regulating chloroplast ribosome biogenesis.

## Results

### Phenotypic characterization of *wfsl1* mutant

**Figure 1:**
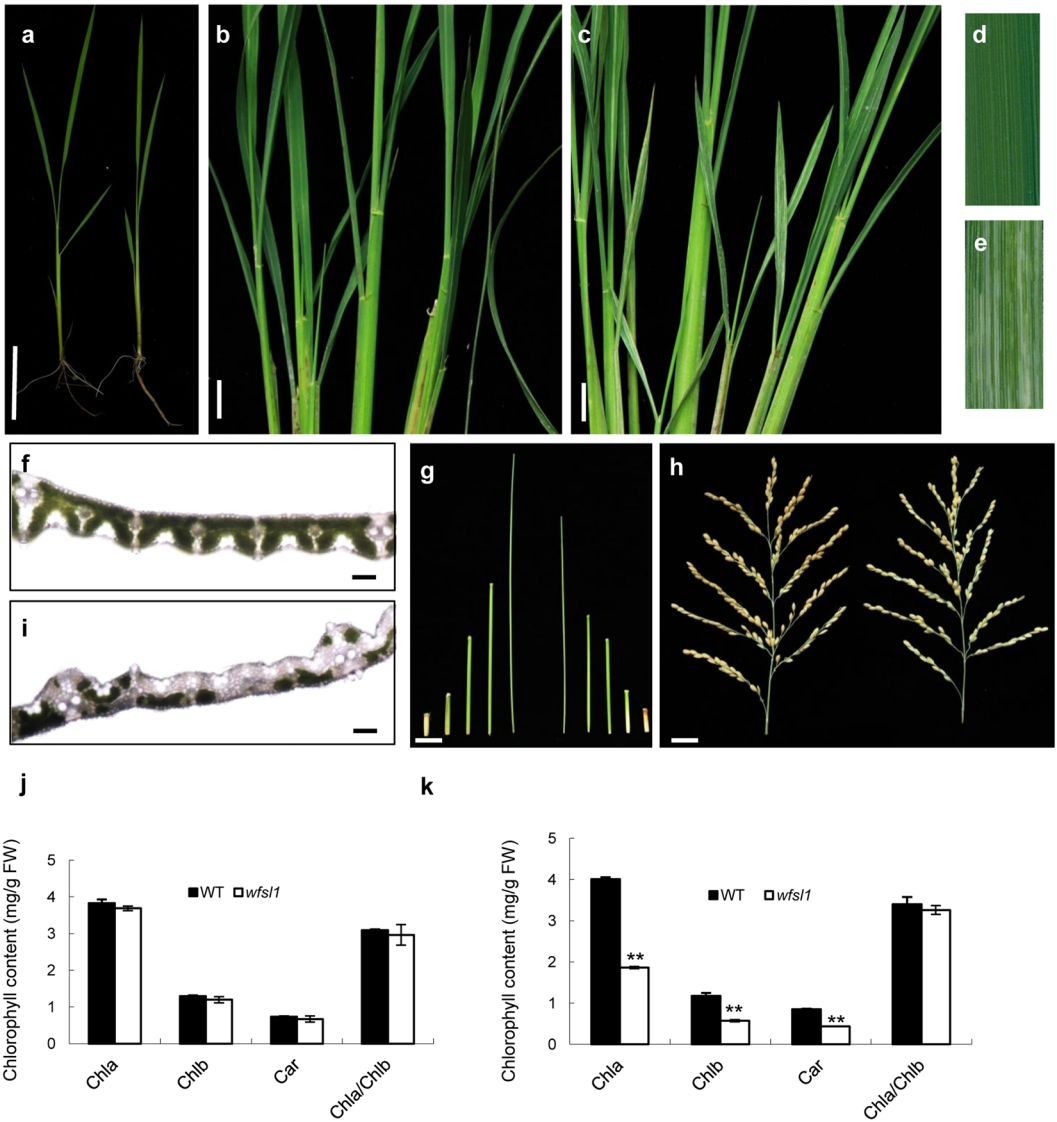
Phenotypes of the wild type and *wfsl1* mutant. (**a**) Wild type and *wfsl1* seedlings. (**b**) Wild type plants at the tillering stage. (**c**) White fine stripe leaf of *wfsl1* at tillering stage. (**d**,**e**) The enlarged leaves of (**b**,**c**). (**f**,**i**) Cross-section of wild type and *wfsl1* leaf at tillering stage. (**g**) The internode length of wild type (left) and *wfsl1* (right). (**h**) The panicle pattern of wild type (left) and *wfsl1* (right). (**j**) The chlorophyll content of wild type and *wfsl1* seedlings. (**k**) The chlorophyll content of wild type and *wfsl1* at tillering stage. Bars: (**a**) 10 cm; (**b**,**c**) 5 cm; (**f**,**i**) 200 μm; (**g**,**h**) 2 cm. Data are mean ± SD (n = 3). Error bars represent the SD from three independent experiments (Student’s *t*-test, *P < 0.05; **P < 0.01).

**Figure 2:**
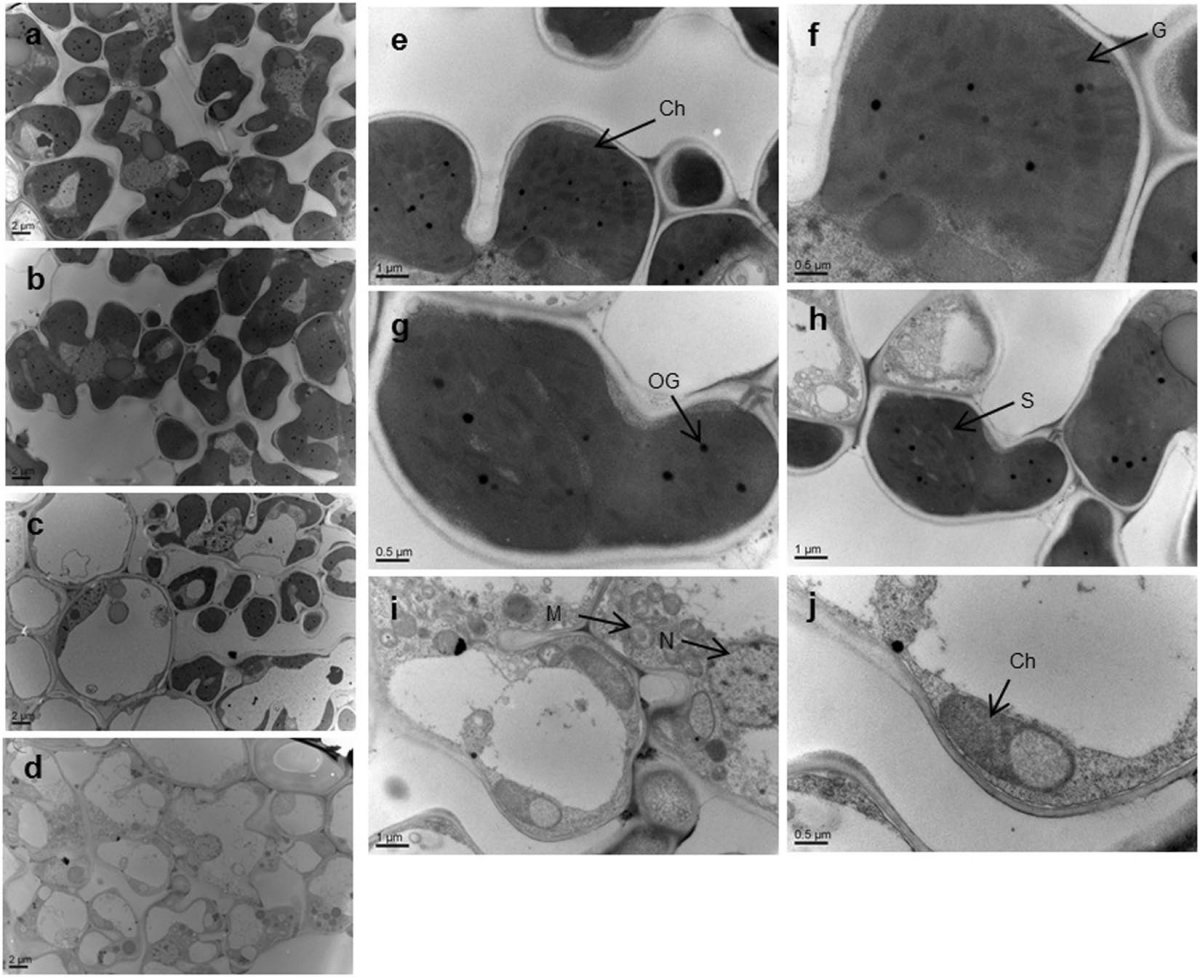
Leaf morphology and transmission electron microscopic images of chloroplasts of wild type and *wfsl1*. (**a**) Wild type. (**b**) Green leaf sectors of *wfsl1*. (**c**) Green and white leaf sectors of *wfsl1*. (**d**) White leaf sectors of *wfsl1*. (**e**,**f**) Wild type leaf sectors chloroplast morphology at tillering stage. (**g**,**h**) Green and white leaf sectors of chloroplast morphology at tillering stage of *wfsl1*. (**i**,**j**) White leaf sectors chloroplast morphology at tillering stage of *wfsl1*.(**Ch**) Chloroplast. (**G**) Grana. (**M**) Mitochondria. (**N**) Nucleus. (**OG**) Osmiophilic plastoglobuli. (**S**) Starch granule.

The *wfsl1* mutant was isolated from an M_2_ population of the *japonica* rice Zhonghua11 (ZH11) after Ethyl methanesulfonate mutagenesis, and was designated according to the chromosome location and white fine stripe leaf phenotype. At seedlings *wfsl1* showed normal phenotype (Fig. 1a) but it emerged white fine stripe leaf at tillering stage and continued to the mature stage (Fig. 1b–e). Cross-section observation showed content of chlorophyll decreased in *wfsl1* mutants (Fig. 1f,i). The chlorophyll and carotenoid levels were nearly the same at seedling stage (Fig. 1j), but they remarkably decreased in *wfsl1* mutants at tillering stage (Fig. 1k). Transmission electron microscopy (TEM) indicated that *wfsl1* had green, green-white and white types ﻿of﻿ cells (Fig. 2a–d). The green cells have more chloroplast and well-developed thylakoid membrane systems, whereas the chloroplast were small with no thylakoid membranes in *wfsl1* white cells (Fig. 2e–j). So the cells are heteroplastidic in mutants.

### The *wfsl1* plants reduced grain yield

**Figure 3:**
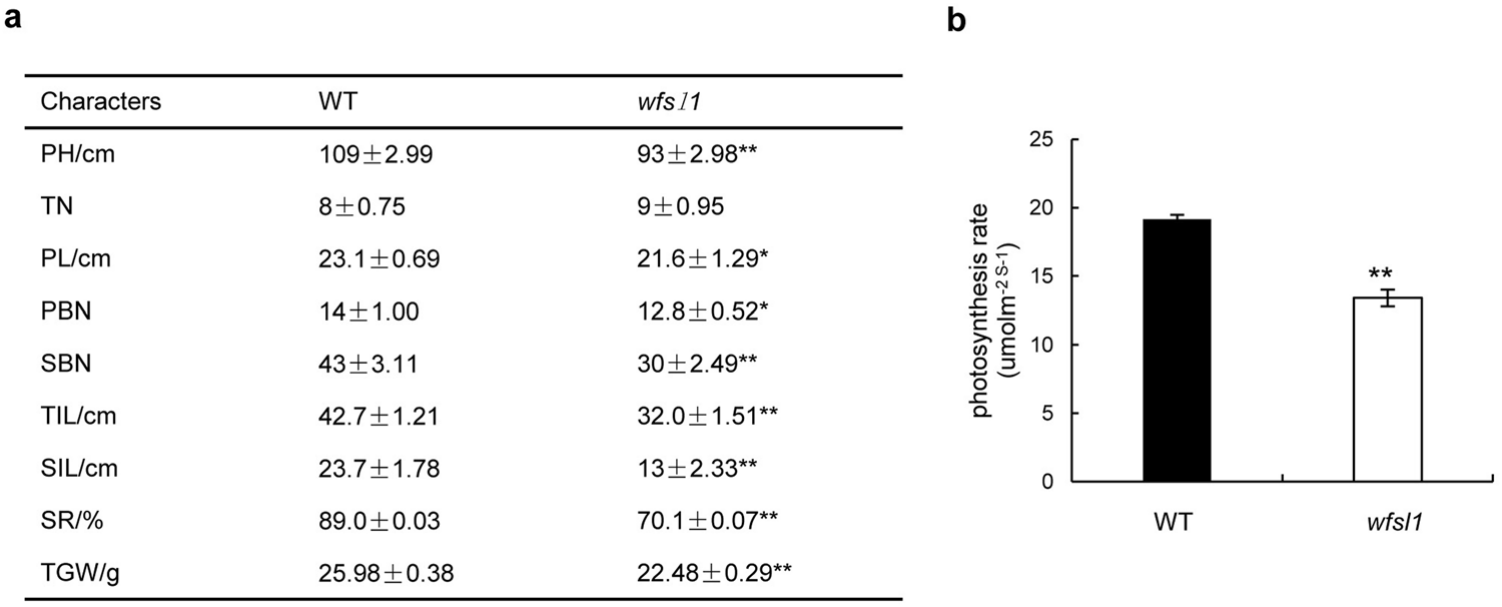
Agronomic traits and photosynthesis analysis of wild type and *wfsl1* plants. (**a**) Agronomic traits analysis of wild type and *wfsl1*. (**b**) Photosynthesis rate analysis of wild type and *wfsl1* at tillering stage. PH Plant height. TN Tiller number. PL Panicle length. PBN Primary branch number. SBN Second branch number. TIL Topmost internode length. SIL Secondmost internode length. SR Setting rate. TGW thousand grain weight. Error bars represent the SD from three independent experiment (Student’s *t*-test, *P < 0.05; **P < 0.01; n = 6).

Agronomic trait analysis indicated that the plant height, panicle length, setting rate, and thousand seed weight were remarkably reduced in *wfsl1*, compared with WT (Fig. 3a). The length of topmost and second topmost interondes was significantly decreased in *wfsl1* (Fig. 3a). The setting rate was 70.1% of *wfsl1* while 89% of wild type (Fig. 3a). The thousand seed weight reduced to 22.48 g of *wfsl1* where wild type was 25.98 g (Fig. 3a). The photosynthesis rate reduced in *wfsl1* (Fig. 3b). However, no difference of tiller number between wild type and *wfsl1* was found (Fig. 3a). TEM suggested chloroplasts were small and thylakoid membrane impaired in *wfsl1* which may reduce photosynthesis. In summary, the *wfsl1* mutation resulted in the reduction of grain yield.

### Map-based cloning and functional confirmation of *WFSL1*

**Figure 4:**
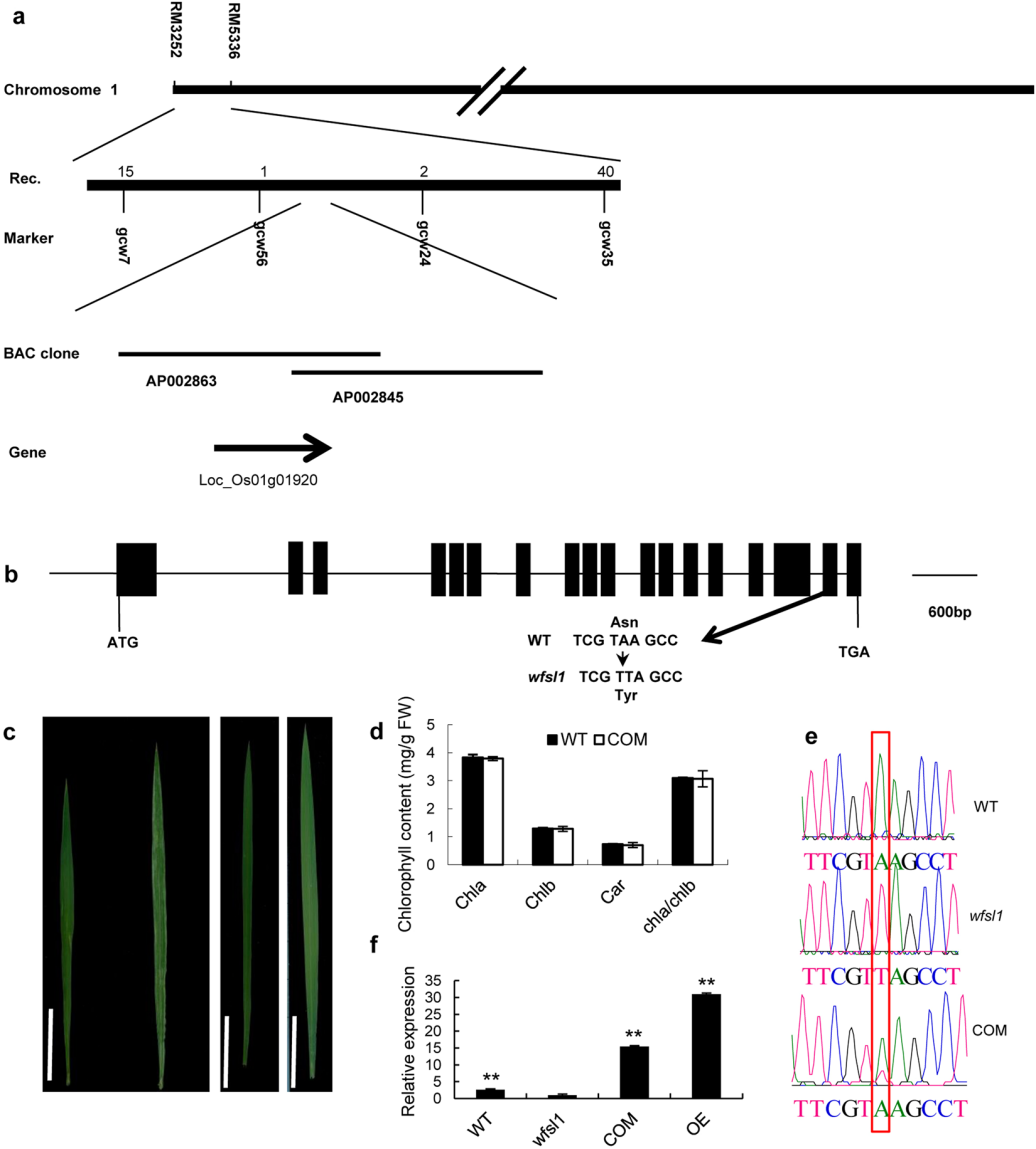
Map-based cloning of the *WFSL1* gene. (**a**) Fine mapping of *WFSL1*. Markers used for mapping are indicated. AP002863 and AP002845 are accession of BACs. (**b**) Structure of *WFSL1*. ATG and TGA represent the start and stop codons, respectively. Black boxes indicate the exons and the lines indicate introns. A single nucleotide replacement ‘A’ to ‘T’ led to an amino acid change. (**c**) Leaf phenotype at tillering stage wild type and *wfsl1* (left), complementation plants (COM) (middle), overexpression plants (OE) (right). (**d**) The chlorophyll content of wild type and complementation plants at tillering stage. (**e**) Sequencing results indicate wild type genome DNA fragment transfer into *wfsl1* plants. (**f**) The expression level of wild type, *wfsl1*, complementation and overexpression at tillering stage. The relative expression level of *wfsl1* represents “1” Data are mean ± SD (n = 3). Bars: (**c**) 5 cm. Error bars represent the SD from three independent experiments.(Student’s *t*-test, *P < 0.05; **P < 0.01)

The *WFSL1* was cloned in an F_2_ population derived from the cross between the *wfsl1* and an *indica* cultivar NJ06. The segregation ratio of wild type to white fine stripe leaf phenotype among the F_2_ population was 3:1 (365:107, *χ* ^*2*^ = 1.3672 < χ^2^_0.05, 1_; *P* = 0.2423 > 0.05), suggesting that the phenotype of *wfsl1* mutant is controlled by a single recessive gene (Supplementary Table S2). The *WFSL1* gene was primarily mapped to the region between the markers RM3252 and RM5336 on chromosome 1 (Fig. 4a). Then eight insertion-deletion polymorphism (InDel) markers were developed for further fine-mapping (Supplementary Table S1). The location of *WFSL1* was narrowed down to a 50.5 kb region which includes 12 ORFs (open reading frame). The predicted region was sequenced to detect the mutation and discovered that the target ORF (LOC_Os01g01920) had an “A” to “T” substitution in protein coding region (Fig. 4a). The gene includes eighteen exons and seventeen introns. The mutation site on the seventeenth exon, with the amino acid Asn changed into Tyr (Fig. 4b). Gene prediction (Rice Genome Annotation Project) indicates that it encodes a putative metal-dependent phosphohydrolase. Sequencing alignment indicates that WFSL1 has a conserved HD domain motif (Supplementary Fig S1). To confirm whether the phenotype of *wfsl1* mutant is caused by the loss function of WFSL1, complementation test was conducted. The genomic DNA fragment containing *WFSL1* sequence, the upstream and downstream sequences were inserted into a binary vector to construct pCAMBIA1300-*WFSL1* vector. This vector was introduced into the *wfsl1* mutants by *Agrobacterium*-mediated transformation. More than eight transgenic lines exhibited normal green leaves (Fig. 4c), and the content of chlorophyll had no difference contrast to wild type (Fig. 4d). Besides, sequencing results confirmed the complementation plants (COM) (Fig. 4e). And a 1,743-bp fragment of full-length cDNA from the cDNA library of wild type was inserted into a binary overexpression vector to construct pCAMBIA1300S-*WFSL1*. The vector was transformed into *wfsl1* mutants to generate overexpression plants (OE). The overexpression plants exhibited normal green leaves and relative expression increased significantly compared with wild type and mutants (Fig. 4f). Thus, the white fine stripe leaf phenotype was rescued. These results suggest that the single base substitution in *LOC_Os01g01920* (*WFSL1*) is responsible for the phenotype of *wfsl1* mutant.

### Location of WFSL1 to chloroplast

**Figure 5:**
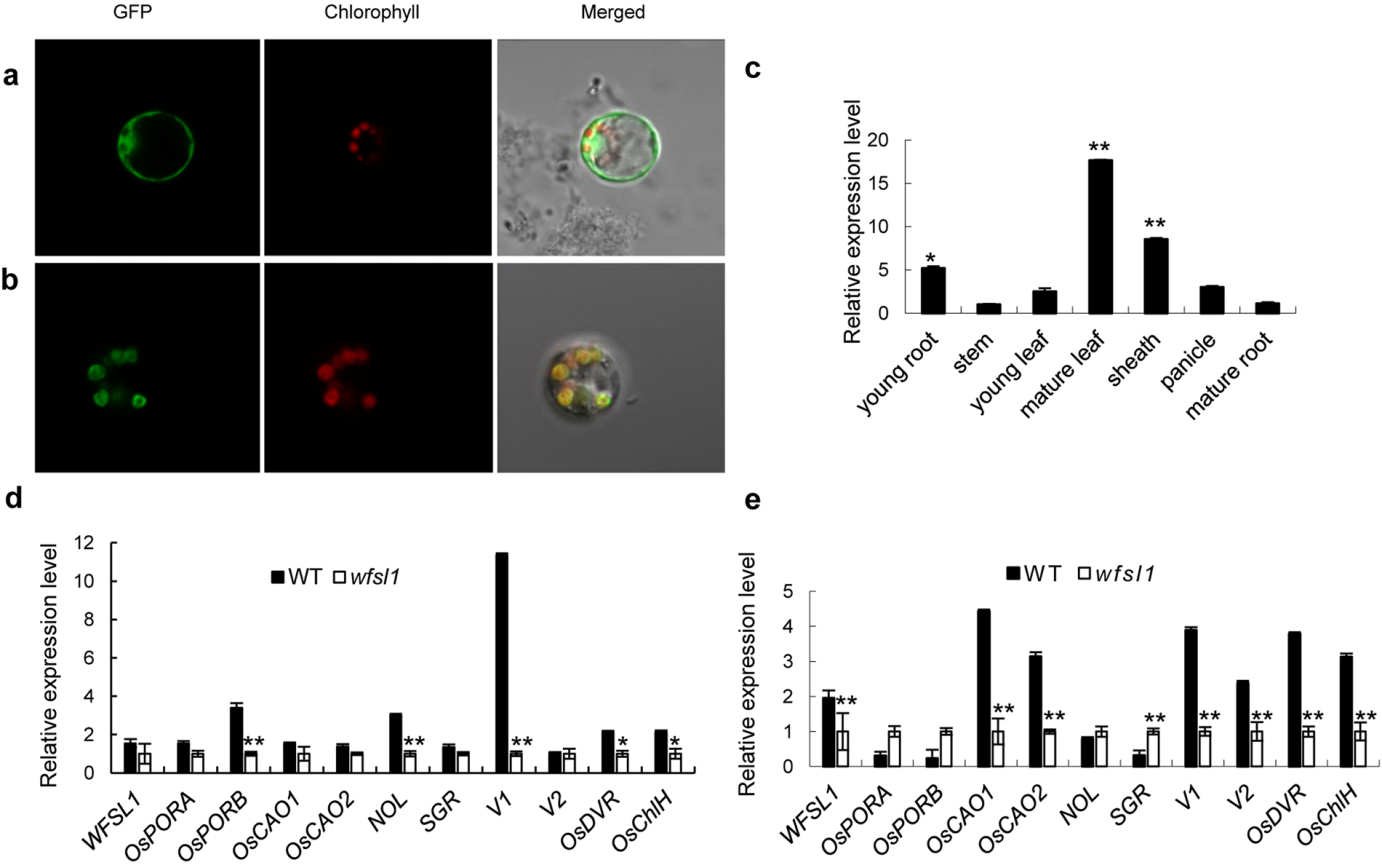
Subcellular localization and expression pattern analysis of *WFSL1*. (**a**,**b**) Localization of GFP (control) and WFSL1 protein in rice protoplasts. Green fluorescence shows GFP, red fluorescence shows chlorophyll, orange indicates the two types of florescence merged. (**c**) qRT-PCR analysis *WFSL1* expression in young root, stem, young leaf, mature leaf, sheath, panicle and mature root of wild type. The relative expression level of stem represents “1”. (**d**,**e**) Relative expression levels of some genes of chloroplast synthesis, degradation and development. Error bars represent the SD from three independent experiments. (Student’s *t*-test, *P < 0.05; **P < 0.01).

To investigate the subcellular localization of WFSL1, the full-length *WFSL1* cDNA was amplified from the cDNA library of wild type. The *WFSL1* cDNA was ligated to *GFP* sequence, and injected into rice protoplasts. Its expression was under the control of 35 S promoter. The GFP fluorescence in transformed protoplasts was examined using confocal fluorescence microscope, which showed that WFSL1 was located to chloroplast (Fig. 5a,b).

### Analysis of *WFSL1* and plastid-encoded genes expression pattern

qRT-PCR was used to examine the tissue-specific expression pattern of *WFSL1*. RNA was extracted from young leaves and young roots (three leaf stage), mature leaves and mature roots (heading stage), stems, sheaths and panicles, respectively. *WFSL1* was highly expressed in sheaths and mature leaves, but low expression in young leaves, roots, stems and panicles (Fig. 5c). Next, we investigated the transcription levels of the genes associated with chlorophyll biosynthesis, chlorophyll degradation or chloroplast development in *wfsl1* mutant. Ten genes were selected, including chlorophyll biosynthesis-related genes *OsPORA* ^21, 22^ and *OsPORB* ^22^, *OsCAO1* and *OsCAO2* ^23^, chlorophyll degradation-related genes *NOL* ^24^ and *SGR* ^25^. Chloroplast developmental genes *V1* ^13–16^, *V2* ^17^, *OsDVR* ^19, 20^ and *OsChlH* ^26^. *UBQ5* (*Actin*) was used as control^27^ (Supplementary Table S3). qRT-PCR analysis showed that the transcriptions of *OsPORB*, *NOL*, *V1*, *OsDVR*, *OsChlH* were significantly suppressed in *wfsl1* mutant at seedling stage. The expression level of *OsPORA*, *OsPORB*, *SGR* were increased and *OsCAO1*, *OsCAO2*, *V1*, *V2*, *OsDVR*, *OsChlH* were decreased in mutant at tillering stage (Fig. 5d,e). The relative expression levels of plastid-encoded genes *PsaA*, *PsbA*, *AtpB* and Rubisco large subunit (*RbcL*), Rubisco activase (*RCA*) were all decreased in *wfsl1*. while the expression levels of nuclear-encoded genes *RpoA*, *RpoB*, *RpoC1*, *RpoC2* increased in *wfsl1*. These results suggest *wfsl1* is defective in biogenesis of plastid-encoded genes and affects chloroplast development.

### Expression of photosynthesis related genes is repressed in *wfsl1*

**Figure 6:**
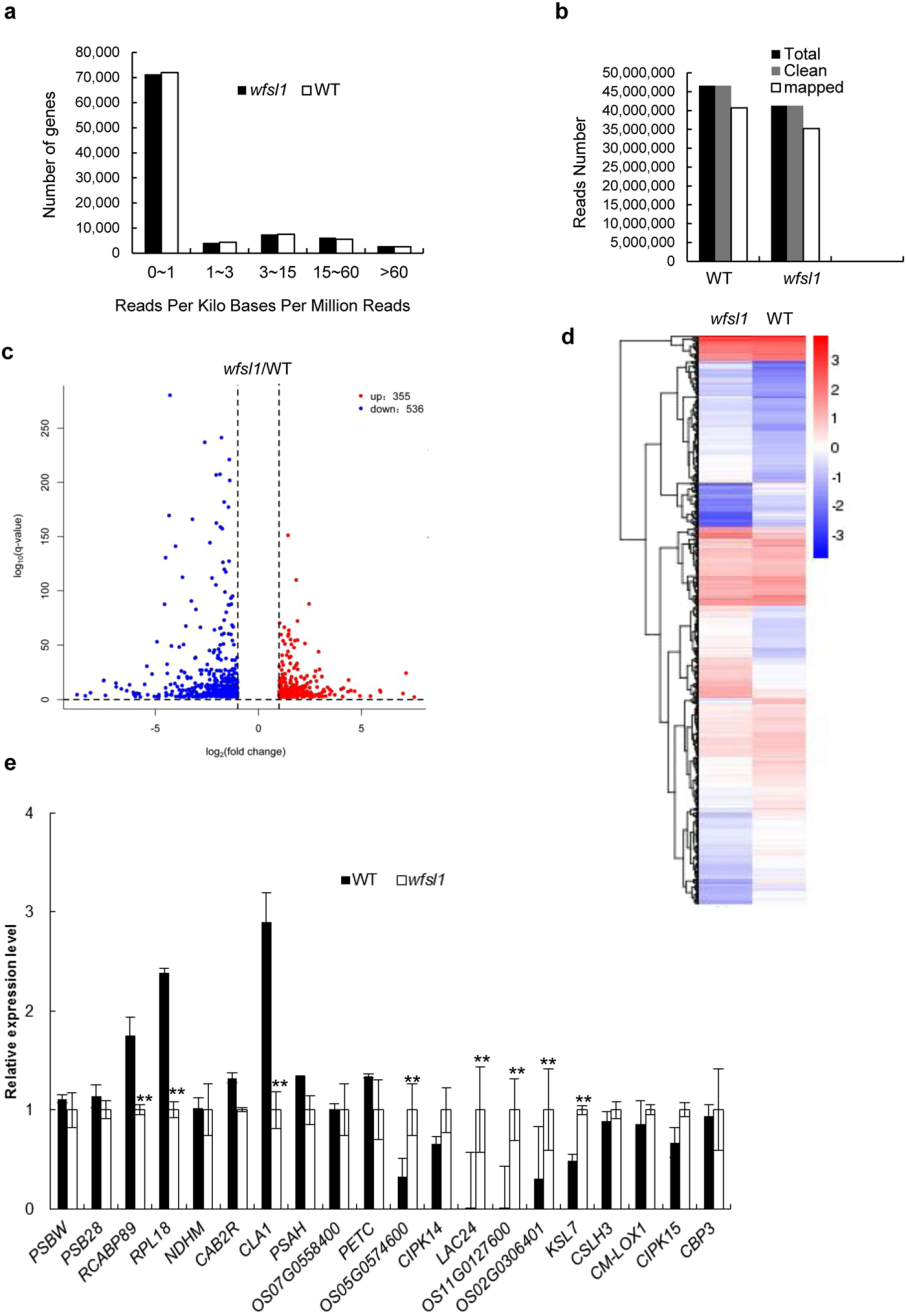
RNA-seq analysis of wild type and *wfsl1*. mRNA was purified from total RNA isolated from tillering stage plants of wild type and *wfsl1* using poly-T oligo-attached magnetic beads. cDNA was synthesized using random hexamer primers. The library was constructed and sequenced using an Illumina Hisequation 2000. (**a**) Numbers of genes sorted according to their expression level. (**b**) Read numbers of wild type and *wfsl1* sequences. (**c**) Volcano plot showing the overall alterations in gene expression in wild type and *wfsl1*. (**d**) Cluster analysis of differently expressed genes in wild type and *wfsl1* Red represents high expression genes. Blue represents low expression genes. (**e**) qRT-PCR analysis differently expression genes of RNA-seq. 20 up-regulated or down-regulated genes were tested. Error bars represent the SD from three independent experiments (Student’s *t*-test, *P < 0.05; **P < 0.01)

RNA-seq was performed to analyze the effect of the *wfsl1* mutation on gene expression. More than 40 million reliable clean reads were obtained from wild type and *wfsl1*. About 355 up regulated genes while 536 repressed genes in *wfsl1* (Fig. 6a–d, Supplementary Table S4). We randomly selected 10 down-regulated and 10 up-regulated genes, and using qRT-PCR methods to verify the results of RNA-seq. The qRT-PCR results were consistent with the RNA-seq (Fig. 6e). Go and KEGG enrichment analysis indicated that genes encoding photosynthesis, light reaction, chloroplast envelope, PSI and PSII, chlorophyll binding, carbon fixation were remarkably reduced in *wfsl1* (Supplementary Figs S2 and S3).

### Defects of WFSL1 and plastid proteins in *wfsl1*

**Figure 7:**
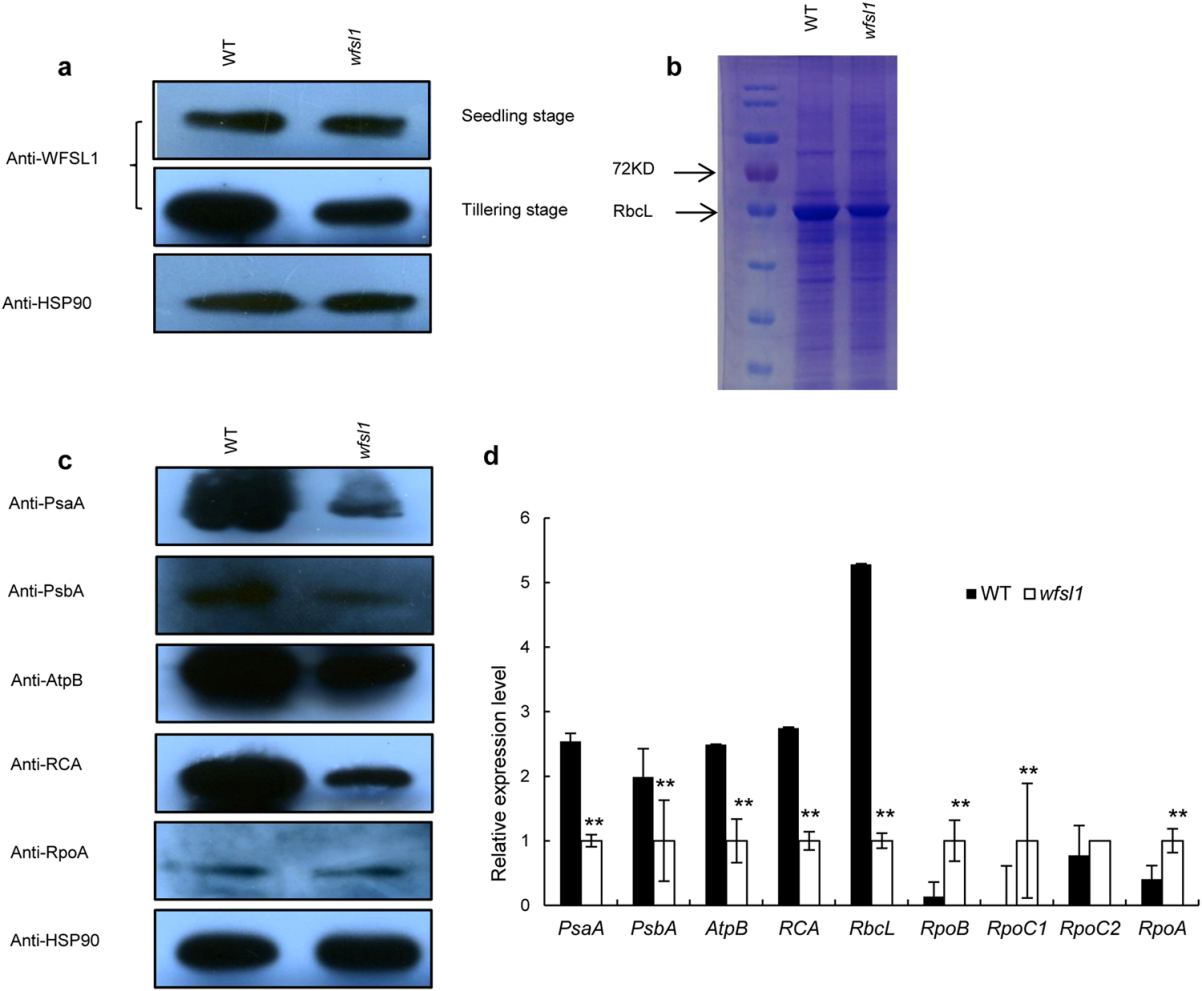
Protein levels analysis of WFSL1 and some representative proteins. (**a**) Western blot analysis of wild type and *wfsl1* at seedling and tillering stage. (**b**) SDS-PAGE gel shows Rubisco using Coomassie Brilliant Blue stained in wild type and *wfsl1* at tillering stage. (**c**) Western blot analysis of chloroplast proteins and RCA. (**d**) qRT-PCR analysis the relative expression level of plastic encoding genes in wild type and *wfsl1* at tilering stage. Hsp90 was used as an internal control. Error bars represent the SD from three independent experiments (Student’s *t*-test, *P < 0.05; **P < 0.01).

**Figure 8:**
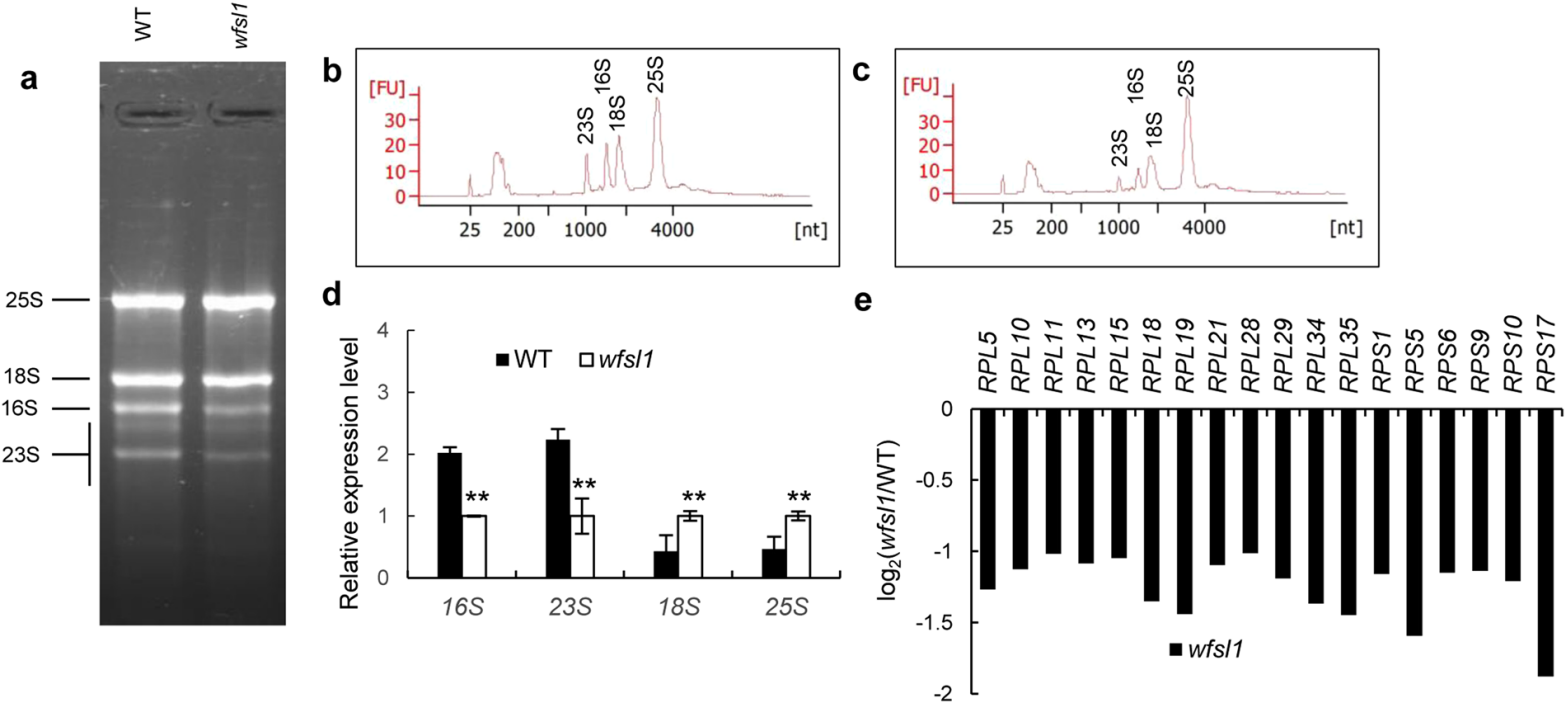
Analysis of rRNA and chloroplast ribosomal gene expression in wild type and *wfsl1*. (**a**) Total RNA from wild type and *wfsl*1 at tillering stage. (**b**,**c**) rRNA analysis from wild type and *wfsl1* using Agilent 2100, respectively. (**d**) qRT-PCR analysis of the expression level of 16 S, 23 S, 18 S and 25 S in wild type and *wfsl1* plants. (**e**) RNA-seq analysis of chloroplast ribosomal genes. RNA was isolated from wild type and *wfsl1* at tillering stage. Error bars represent the SD from three independent experiments (Student’s *t*-test, *P < 0.05; **P < 0.01).

We tested the accumulation of WFSL1 protein in wild type and *wfsl1* mutants using western-blot analysis. However, the accumulation of WFSL1 protein was decreased in *wfsl1* mutant at tillering stage (Fig. 7a). SDS-PAGE gel and western-blot suggest the protein levels of the large subunit of Rubisco (RbcL) and Rubisco activase (RCA) were decreased in *wfsl1* (Fig. 7b,c).Other plastidic proteins including ATP synthase subunit beta (AtpB), A1 of PSI, D1 of PSII, “alpha and beta” subunits of RNA polymerase were also tested. The results showed that the levels of plastid-encoded proteins were significantly decreased in *wfsl1* (Fig. 7c). qRT﻿-PCR results suggest the expression levels of class I genes *RbcL*, *PsbA*, *AtpB* were remarkably decreased, while class III genes including *RpoA*, *RpoC2* increased (Fig. 7d). RNA-seq results indicated that expression levels of many plastidic genes changed between wild type and *wfsl1*. Class I, II and III genes are three types of plastidic genes in plant. Class I genes are transcribed by PEP, class II genes are transcribed by NEP and PEP, and class III genes are mainly transcribed by NEP. The expression levels of class III genes were increased while class I genes decreased (Fig. 8d). These results indicate that *wfsl1* was defective in PEP activity and chloroplast protein biosynthesis.

### Reduction of chloroplast ribosome activity in *wfsl1*

Chloroplast ribosome is composed of 30 S small subunit and 50 S large subunit. The 30 S and 50 S subunits are mainly comprised of 16 S and 23 S rRNAs and ribosomal genes^9^. We found that the 16 S and 23 S rRNAs were decreased in *wfsl1* (Fig. 8a). We used an Agilent 2100 to analyze the composition and content of rRNAs from wild type and *wfsl1* at tillering stage. We found that the 16 S and 23 S rRNAs were reduced to one half of wild type levels (Fig. 8b–d). RNA-seq analysis indicates that the expression levels of some ribosomes 30 S genes *RPS1*, *RPS5*, *RPS17* and 50 S genes *RPL5*, *RPL11*, *RPL13*, *RPL18* were all decreased in *wfsl1* (Fig. 8e). These results indicated that *wfsl1* mutant was defective in biogenesis of chloroplast ribosomes.

## Discussion

Rice leaf-color mutants are key for investigation of chloroplast development, light energy utilization rate and reproduction. In this study, we isolated the *wfsl1* mutant displayed white fine stripe leaves at tillering stage (Fig. 1b,c). Compared with the wild type, *wfsl1* mutant exhibited pleiotropic phenotypes including reduction of plant height, panicle length, seed setting rate, and thousand seed weight. Also the chlorophyll content decreased in *wfsl1* plants since tillering stage compared with WT (Fig. 1f,i,j,k). *wfsl1* has three types of cells (green, green and white, white) and its chloroplasts were defective in tillering stage (Fig. 2e–j). TEM results identified the pattern of the white fine stripe phenotype, suggesting that *wfsl1* cells are heteroplastidic containing white cell sector with abnormal chloroplasts (Fig. 2a–d). The *wfsl1* mutant only has single-base mutation and leads to a series of phenotypic changes. These phenotypic differences between the *wfsl1* and previously reported mutants may be due to the different genetic backgrounds and the different mutation sites in the target genes^28–30^. So determination of the pattern of white fine stripe leaf using the *wfsl1* mutant is an important to understand the mechanism of white fine stripe and the function of WFSL1 protein. The reduction of the content of chlorophyll in *wfsl1* is useful to study the relationship among WFSL1 and chloroplast development, light energy utilization and grain yield.

We cloned *WFSL1* using map-based cloning method and confirmed its function in transgenic plants (Fig. 4). WFSL1 has a HD domain motif^31^ (Supplementary Fig. S1) with a H…HD…H…H…D sequence motif. The histidines or aspartates in HD domain are highly conserved and coordinate with the metal ion to regulate the activity of protein. HD domain is globular in dGTPases^32^ and has the (Guanosine 5′-triphosphate 3′-diphosphate) ppGpp hydrolase activity of SpoT protein in *E*.*coli*, which supports that the protein containing HD-domain is a phosphohydrolase^33^. Cyclic nucleotide Phosphodiesterase (PDEs) has a HD domain, and is regulated by Zn^2+^ coordination. Other metals, such as Mn^2+^, Co^2+^ and Mg^2+^, also have catalytic activity^34^. *RelA* and *SpoT* regulate ppGpp levels in *E*.*coli* ^35^. In SpoT protein, HD domain has ppGpp_ase_ activity^33^, and the His-Asp doublet is involved in the hydrolysis for ppGpp^36^. *Arabidopsis* has four RelA/SpoT homologues *At*RSH1, *At*RSH2, *At*RSH3 and *At*CRSH. They are all targeted to plastids and expressed in green tissues and flowers which have important functions in chloroplast development and reproduction via ppGpp synthetase activity^37, 38^. The transcripts encoding the RC and LHC subunits of PSI and PSII, the small and large subunits of Rubisco was decreased in OX:RSH2 and OX:RSH3 plants. Plastid-encoded genes were affected in OX:RSH2 and OX:RSH3 plants^39^.

Also WFSL1 was located to chloroplast (Fig. 5a,b), and the same that the HD domain containing proteins OsCRSH1, OsCRSH2 and OsCRSH3 were located to chloroplast^38^. Go term analysis found that genes of photosynthesis, photosystem II assembly, and plastid were highly expressed in *wfsl1* (Supplementary Fig. S1). KEGG analysis found that photosynthesis, and carbon fixation metabolism were significantly difference in WT and *wfsl1* (Supplementary Fig. S2). These suggest WFSL1 was essential in chloroplast development. The expression of chlorophyll synthesis genes *OsCAO1* and *OsCAO2*, chloroplast development genes *V1*, *V2*, *OsDVR* and *OsChlH* and plastid encoded genes *PsaA*, *PsbA*, *AtpB*, *RCA* were all decreased in *wfsl1* at tillering stage. The expression level of nuclear-encoded genes *RpoA*, *RpoB*, *RpoC1*, *RpoC2* was increased in *wfsl1* (Fig. 7d). These suggest plastid encoded genes were defective in *wfsl1*. *WFSL1* is highly expressed in mature leaves and sheaths, indicating that it functions at tillering and mature stages (Fig. 5d).

The expression class I genes decreased and class III genes increased in *wfsl1*. It is similar to these PEP-related mutants *obgc* ^8^, *rh3* ^40^, and PPR proteins mutants^41, 42^. So we further conducted western blot analysis, and found WFSL1 protein levels were decreased in *wfsl1* at tillering stage (Fig. 7a). These results in decreased expression levels of ﻿*WFSL1* at tillering stage (Fig. 5h,i). The rubisco large subunit, plastid-encoded proteins and Rubisco activase (RCA) were decreased in *wfsl1*. Nuclear-encoded protein RpoB increased in *wfsl1*. qRT-PCR results indicate that the expression levels of plastid-encoded genes decreased and nuclear-encoded genes increased in *wfsl1*. These are very likely previously reported mutant *wp1* ^9^. The *wp1* mutant decreased plastid-encoded proteins and defective in chloroplast developmental. Since levels of plastid-encoded proteins decreased in *wfsl1*. We analyzed the content and composition of rRNA using an Agilent 2100. The results showed little difference between 18 S and 25 S rRNA levels in wild type and *wfsl1*. However, we found that *wfsl1* 16 S and 23 S rRNAs contents were dropped one half that of wild type (Fig. 8a–d). RNA-seq analysis suggest expression levels of ribosomal genes, including 50 S ribosomal genes *RPL5*, *RPL10*, *RPL18*, *RPL21* and 30 S ribosomal genes *RPS1*, *RPS5*, *RPS9*, *RPS17* were all decreased in *wfsl1* (Fig. 8e). These results indicated that *wfsl1* was defective in chloroplast ribosome biogenesis

Chloroplast plays an important role in light reception in higher plants^1^. In this study, the *wfsl1* displayed white fine stripe leaves and reduced Chl content at tillering stage (Fig. 1f,i,k). The thylakoid was abnormal in *wfsl1* (Fig. 2e–j), and leads to the reduced of photosynthesis rate. Some plastid-encoded proteins A1 of PSI (PsaA), D1 of PSII (PsbA), Rubisco large subunit and Rubisco activase (RCA) were decreased in *wfsl1* (Fig. 7a–c). PSI and PSII are two pathways that responsible for electron transfer during photosynthesis. Ribulose-1,5-bisphosphate (RuBP) carboxylase/oxygenase (Rubisco), which constitute of RbcL and RbcS subunit that catalyzes the first step in net photosynthetic CO_2_ assimilation and photorespiratory carbon oxidation^43, 44^. PSI and PSII may inhibit protein levels of RbcL in *wfsl1*, suggesting that the photosynthesis was affected in *wfsl1* plants. The decreased setting rate and thousand seed weight (Fig. 3a) were associated with decreased photosynthesis in *wfsl1* plants. These results showed that defective developmental of chloroplast affected the photosynthesis rate and grain yield in *wfsl1*.

In conclusion, this study suggested that WFSL1, which is essential in expression of plastid genes and plastid ribosome biogenesis, is important for chloroplast development.

## Materials and Methods

### Plant materials and growth conditions

The *wfsl1* mutant was isolated from a M_2_ population of the *japonica* rice ZH11 (wild type) after Ethyl methanesulfonate mutagenesis. The *japonica* rice ZH11 and *wfsl1* mutant were grown in the paddy fields of Zhejiang (30°03′N, summer season, temperate climate) and Hainan (18°48′N, winter season, subtropical climate) provinces in China under local growing conditions.

### Chlorophyll analysis

The leaves from different stages of wild type and *wfsl1* were collected and weighted. Then, the leaves were soaked in an acetone and ethanol mixture solution at 26 °C in dark for 24 h. The content of chl *a*, chl *b* and carotenoids were calculated as described previously^45^.

### Transmission electron microscopy (TEM) analysis

The leaves of wild type and *wfsl1* were selected at tillering stages and cut into small pieces. They were fixed by 2.5% glutaraldehyde (PH 7.2) and vacuumed until fully sinking to the bottom. Subsequently, samples were successively washed three times with 0.2 mol/L sodium cacodylate buffer for 30 min, fixed in 10% osmic acid for 1 h, distilled three times with deionized water for 45 min, dehydrated with ethanol, treated with acetone and embedded in epoxy resins and polymerized at 70 °C. The samples were then cut into about 500–800 Ǻ thick with a slicer and stained by the mixture of uranyl acetate dihydrate and lead citrate. The sections were washed with deionized water and visualized using HITACHI Transmission Electron Microscope (HT7700).

### Genetic analysis and map-based cloning

For genetic analysis, we constructed the crosses between *wfsl1* and the *indica* cultivars TN1, 93–11, SH527 (Shuhui527) to analyze whether a dominant/recessive single/multiple gene control(s) the *wfsl1* phenotype. The segregation population of F_2_ was examined by *χ* ^*2*^ test (Additional file 1: Table S2).

To map the genomic location of *WFSL1*, 1,900 mutants were selected from the F_2_ population which was derived from the cross between *wfsl1* and the *indica* cultivar NJ06 (Nanjing06). A total of 117 pairs of rice chromosome markers were used for primary mapping, and the sequence-tagged-site (STS) markers for fine mapping were developed based on the gap difference between the contig sequences of the *japonica* cultivar Nipponbare and the *indica* cultivar 93–11 (http://ensemblgenomes.org). *WFSL1* was ultimately mapped to a 50.5 kb region on chromosome 1. The predicted region was PCR-amplified and sequenced to detect the mutation.

### Sequence alignment of WFSL1 protein

Using blastp program to search the protein sequence database at the NCBI with an *E*-value cut-off of 0.001. Using the Clustal Omega (http://www.ebi.ac.uk/Tools/msa/clustalo/) to identify motif that are conserved in the alignment sequence.

### Complementation test and overexpression

A 11,296 bp genomic DNA fragment containing *WFSL1* sequence, the upstream and downstream sequences was amplified by two pairs of PCR primers: 5′-ggatcccttgggtgtgccgtcgatgtgagc-3′, 5′-tatccacaacagtgaaaggatatgtggttaac-3′ and 5′-gcattgtgtcattcaggctgcgggatctaaaac-3′, 5′-ccaagcttcggtggtaggaggactcccgttgg-3′. The 11,296 bp PCR product was inserted into the binary vector pCAMBIA1300 (containing CaMV 35 S promoter) to construct the pCAMBIA1300-*WFSL1* vector. This vector was then introduced into the *wfsl1* mutants by *Agrobacterium*-mediated transformation using the *Agrobacterium tumefacien* EHA105. For the *WFSL1* overexpression construct, a 1,743-bp fragment of full-length cDNA was amplified by two pairs of PCR primers: 5′-GGGGTACCATGAAACATCCCTCCCGCATTAAATTGGC-3′ and 5′-GCTCTAGATCAGTTGTAGGTTCTCGAAGGCTTCTG-3′ from the cDNA library of wild type and inserted into the binary overexpression vector pCAMBIA1300S (pCAMBIA1300 containing 35 S promoter) into pCAM-BIA1300S-WFSL1. The vector was transformed into *wfsl1* mutants to generate overexpression plants.

### Identifying the subcellular location of WFSL1

To investigate the subcellular location of WFSL1, a 1,743-bp fragment of full-length cDNA was amplified by PCR and ligated into pCAMBIA1300-GFP (containing 35 s promoter and GFP reporter protein) vector to generate the pCAMBIA1300-WFSL1-GFP construct. The PCR primers for *WFSL1* were: 5′-gctctagaatgaaacatccctcccgcattaaattggc-3′ and 5′-cgggatcccggttgtaggttctcgaaggcttctg-3′. The pCAMBIA1300-WFSL1-GFP vector, as well as the control were transformed into rice protoplasts according to the protocol described previously^46^. The transformed rice protoplasts cells were incubated for 16 h at 28 °C under dark conditions. The GFP fluorescence in transformed protoplasts was examined by confocal fluorescence microscope (Carl Zeiss, LSM 780).

### RNA extraction and qRT-PCR

Total RNA was extracted from the seedling and tillering stage of wild type and *wfsl1* mutants using a Total RNA Extraction Kit (Axygene, cat No, AP-MN-MS-RNA-250) according to the manufacturer’s instructions. The complementary DNA was synthesized using a ReverTra Ace qPCR-RT Kit (TOYOBA, Japan). RT-PCR was run in Applied Biosystems 7900HT Real-time System using 2 × SYBR Green PCR Master Mix (Applied Biosystems). The RT-PCR program was as follows: initial denaturation at 95 °C for 10 min, followed by 40 cycles at 95 °C for 10 s and 60 °C for 1 min. The analysis of each sample was based on three technical replicates and biological replicates. The relative expression level of each transcript was compared with that of *UBQ5* and quantified with the 2^−ΔΔ*C*^_T_ method^47^. The primers for the genes are listed (Supplementary Table S3).

### RNA-seq analysis

Total RNA was extracted from wild type and *wfsl1* at tillering stage. mRNA was purified from total RNA using poly-T oligo-attached magnetic beads. cDNA was synthesized using random hexamer primers. The library was constructed and sequenced using an Illumina Hisequation 2000 (Novogene). A total of 45 million reads genes from wild type and 40 million from *wfsl1* were obtained. The significance of differentially expressed genes (DEGs) were using log_2_ (fold change) > 1 and q values < 0.05. Gene ontology analysis was performed on GOseq^48^. Pathway enrichment analysis was using the Kyoto Encyclopedia of Genes and Genomes database^49^.

### Western blot analysis

Total proteins extraction was performed as previously described^50^ isolated from wild type and *wfsl1* at seedling and tillering stage. The tissues were ground in liquid nitrogen and thawed in extraction buffer [50 mM Tris–HCl pH 7.5, 150 mM NaCl, 10% glycerol (v/v), 0.1% Nonidet P-40, 1 mM DTT, 1 mM PMSF, and 1x complete protease inhibitor cocktail (Roche)] for 15 min on ice. The supernatant was collected by centrifugation at 12,000 g for 10 min at 4 °C. Total proteins were separated by SDS-PAGE gels (8%), transferred to the polyvinylidene difluoride (PVDF) membranes (GE Healthcare), blotted with different primary antibodies, detected with ECL prime (GE Healthcare). Anti-WFSL1 antibody was obtained from Shanghai Youke Biotechnology (http://www.youke-ab.cn/) and other antibodies such as anti-PsaA (LOC_Osp1g00340.1, Cat:AbP80033-A-SE), anti-AtpB (LOC_Os10g21266.1,Cat:AbP80331-A-SE), anti-RCA (LOC_Os11g47970.1, Cat:AbP80246-A-SE) and anti-RpoA (LOC_Osp1g00660.1, Cat:AbP80103-A-SE) from Beijing Protein Innovation (http://www.proteomics.org.cn/) and anti-PsbA (LOC_Osp1g00110.1, Cat:AS05084) from Agrisera.

## Electronic supplementary material

Supplementary information
