# Supplementary material for: Single-point Mutation of an Histidine-aspartic Domain-containing Gene involving in Chloroplast Ribosome Biogenesis Leads to White Fine Stripe Leaf in Rice
Source: Sci Rep. 2017 Jun 12;7:3298. doi: 10.1038/s41598-017-03327-2 (PMC5468306; doi:10.1038/s41598-017-03327-2)
Supplement: Supplementary file 1 — Supplementary information [file 41598_2017_3327_MOESM1_ESM.pdf]

# Single-point Mutation of an Histidine-aspartic Domain-containing Gene involving in Chloroplast Ribosome Biogenesis Leads to White Fine Stripe Leaf in Rice

Changwei Ge[a,b,1], Li Wang[a,1], Weijun Ye[a], Liwen Wu[a], Yongtao Cui[a], Ping Chen[a], Jiangjie Pan[a,b], Dong Zhang[b], Jiang Hu[a], Dali Zeng[a], Guojun Dong[a], Qian Qian[a], Longbiao Guo[a, *], Dawei Xue[b, *]

[a]State Key Laboratory of Rice Biology, China National Rice Research Institute, Hangzhou 310006, China; [b]College of Life and Environmental Sciences, Hangzhou Normal University, Hangzhou 310036, China;

* Correspondence: guolongbiao@caas.cn; dwxue@hznu.edu.cn

[1]Equal contributors

**Supplementary information includes Figure S1-S5 and Table S1-S4**

## Supplementary Figures

```
WFSL1                       -TKQVFDNLHGNISLD---PLAREFVDTEEFQRLRDLKQLGLTYLVYPGAVHTRFEHSLG
Y461_MYCGE:8-268            ------DPILGEIIFDENTKWMYELVNTKAFQRLRNIKQLGINFHFYPSGVHTRYAHSLG
Y677_MYCPN:2-268            QQIFFKDPILGEVLFDQQTKWMYELVETEAFRRLRNIKQLGINFHFYPGGVHTRYSHSLG
YWFO_BACSU:7-337            EEKVFKDPVHRYVHVRD--KLIWDLIGTREFQRLRRIKQLGTTYLTFHGAEHSRFNHSLG
YS48_CAEEL:46-495           ----INDNVYGTVKVP---RPIDKLIDTVEFQRLRHLKQTGLVYLVYPNCEHSRFVHSLG
Y1154_METJA:2-272           --KVIRDSIHKDIYLD---EKELEIIDSEEFQRLRNIKQTGLTYLVYPSANHTRFEHSLG
YL394_MIMIV:22-347          -------NIYGFIRVT---SMAQKIIDTSEFQRLRNMKQLGLCYLVFPAATHTRLEHSIG
YDHJ_BACSU:2-88             ---EISDIIYGQHHID---GVLEELIKSAPVQRLKGIYQGGASFLVNRKWNVTRYEHSIG
SAMH1_DICDI:76-442          SSKIINDVIHGHMEVP---DYIMDFIDTEQFQRLRDLKQVGTTSFVFPCASHSRFEHSIG
SAMH1_DANRE:99-500          ------DPIHGHIELH---PLLLHFIDTPQFQRLRHIKQLGGTYLVFPGASHNRFEHSIG
SAMH1_MOUSE:112-532         LMKVFNDPIHGHIEFH---PLLIRIIDTPQFQRLRYIKQLGGGYYVFPGASHNRFEHSLG
SAMH1_BOVIN:102-510         TMKVINDPIHGHIEFH---PLLMRIIDTPQFQRLRYIKQLGGGYYVFPGASHNRFEHSLG
SAMH1_HUMAN:114-521         TMKVINDPIHGHIELH---PLLVRIIDTPQFQRLRYIKQLGGGYYVFPGASHNRFEHSLG
SAMH1_CHICK:107-513         --KVFNDPVHGHIEIH---PLLVRIIDTPQFQRLRYIKQLGGTYFVFPGASHNRFEHSLG
                                                                                    H

WFSL1                       VYWLAGEAMNNLRLY-----------QGEELGIDRVDMQTVKLAGLLHDIGHGPFSHLFE
Y461_MYCGE:8-268            VYELIRRILNSS----------------AFLNIDQIKKQTVLVAGLLHDLGHGPHSHAFE
Y677_MYCPN:2-268            VYELLRRILNTP----------------AFAPIDENKKQTVLVAGLLHDIGHAPHSHAFE
YWFO_BACSU:7-337            VYEIVRRMVDDVFKGR------------PEWDDSER--ELCLAAALLHDLGHGPFSHSFE
YS48_CAEEL:46-495           TFSLAYALVDKLRHS-----------Q-PSLNITESDLICTSVAALLHDVGHGPFSHLFD
Y1154_METJA:2-272           TMFIASKIAEKINAD----------------------VELTRVSALLHDIGHPPFSHTLE
YL394_MIMIV:22-347          VYDRTRKVIERIYRQYPDREYYIPELSDKPIKLDAKIIECIKIAGLCHDIGHGPFSHVFD
YDHJ_BACSU:2-88             VMLLIKKLGGT---------------------------IEEQIAGLLHDVSHTAFSHVVD
SAMH1_DICDI:76-442          VSHLAGKYIDRIKVT-----------Q-PELEITEREQKFVRIAGLCHDLGHGPFSHAFE
SAMH1_DANRE:99-500          VGYLAGCLVKALNER-----------Q-PELFITKQDILCVQIAGLCHDLGHGPFSHMFD
SAMH1_MOUSE:112-532         VGYLAGCLVRALAEK-----------Q-PELQISERDILCVQIAGLCHDLGHGPFSHMFD
SAMH1_BOVIN:102-510         VGYLAGRLVRELSEK-----------Q-PELQISERDILCVQIAGLCHDLGHGPFSHMFD
SAMH1_HUMAN:114-521         VGYLAGCLVHALGEK-----------Q-PELQISERDVLCVQIAGLCHDLGHGPFSHMFD
SAMH1_CHICK:107-513         VGYLAGCLVRELKER-----------Q-PELDITQRDILCVEIAGLCHDLGHGPFSHMFD
                                                                               HD  H    H
```

**Supplementary Figure S1. WFSL1 protein alignment.** Using blastp program to search the protein sequence database at the NCBI with an E-value cut-off of 0.001. Using the Clustal Omega (http://www.ebi.ac.uk/Tools/msa/clustalo/) to identify motif that are conserved in the alignment sequence. The conversed HD domain motif are indicated with blank font.

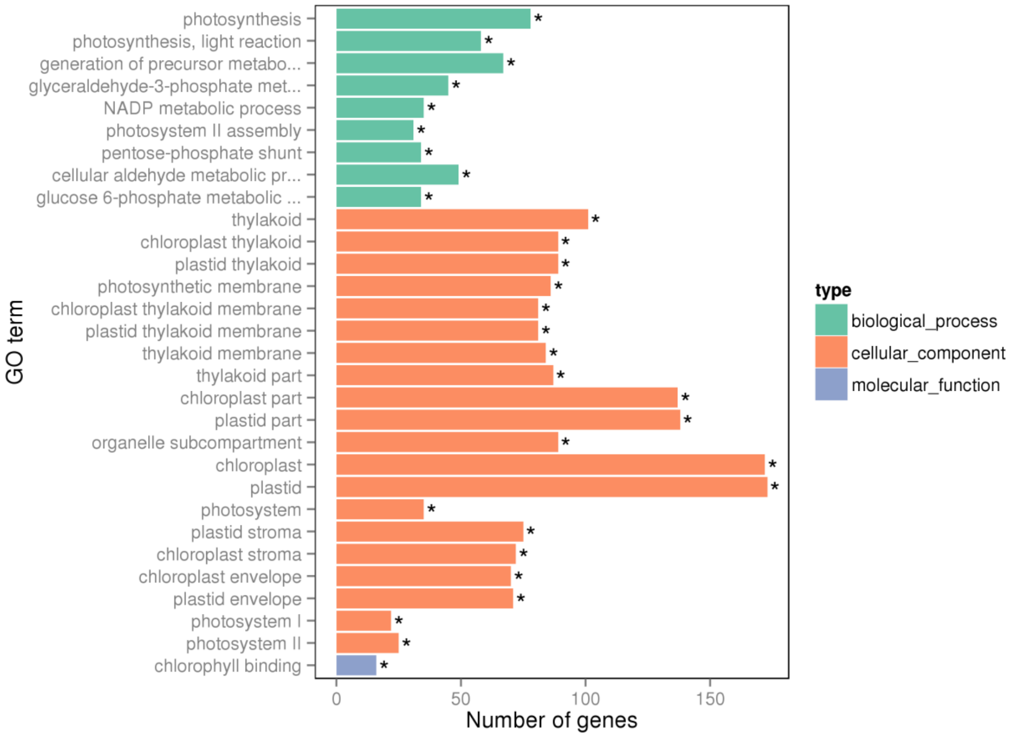


**Supplementary Figure S2. Go enrichment analysis of differently expressed genes in wild type and *wfsl1*.** Go terms with *P-value* less than 0.05 were considered significantly enriched by differential expressed genes in wild type and *wfsl1*.

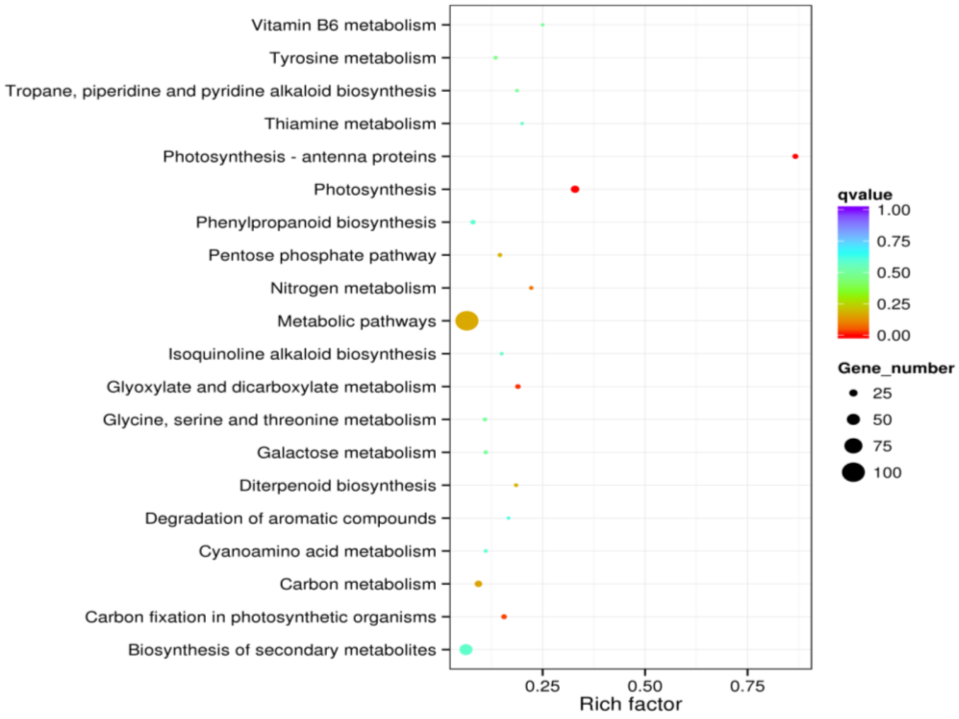


**Supplementary Figure S3. KEGG enrichment analysis of differently expressed genes in wild type and *wfsl1*.** KEGG pathways used KOBAS software to test the enrichment of differential expression genes in wild type and *wfsl1*.

**Supplementary Figure S4 Western Blot**

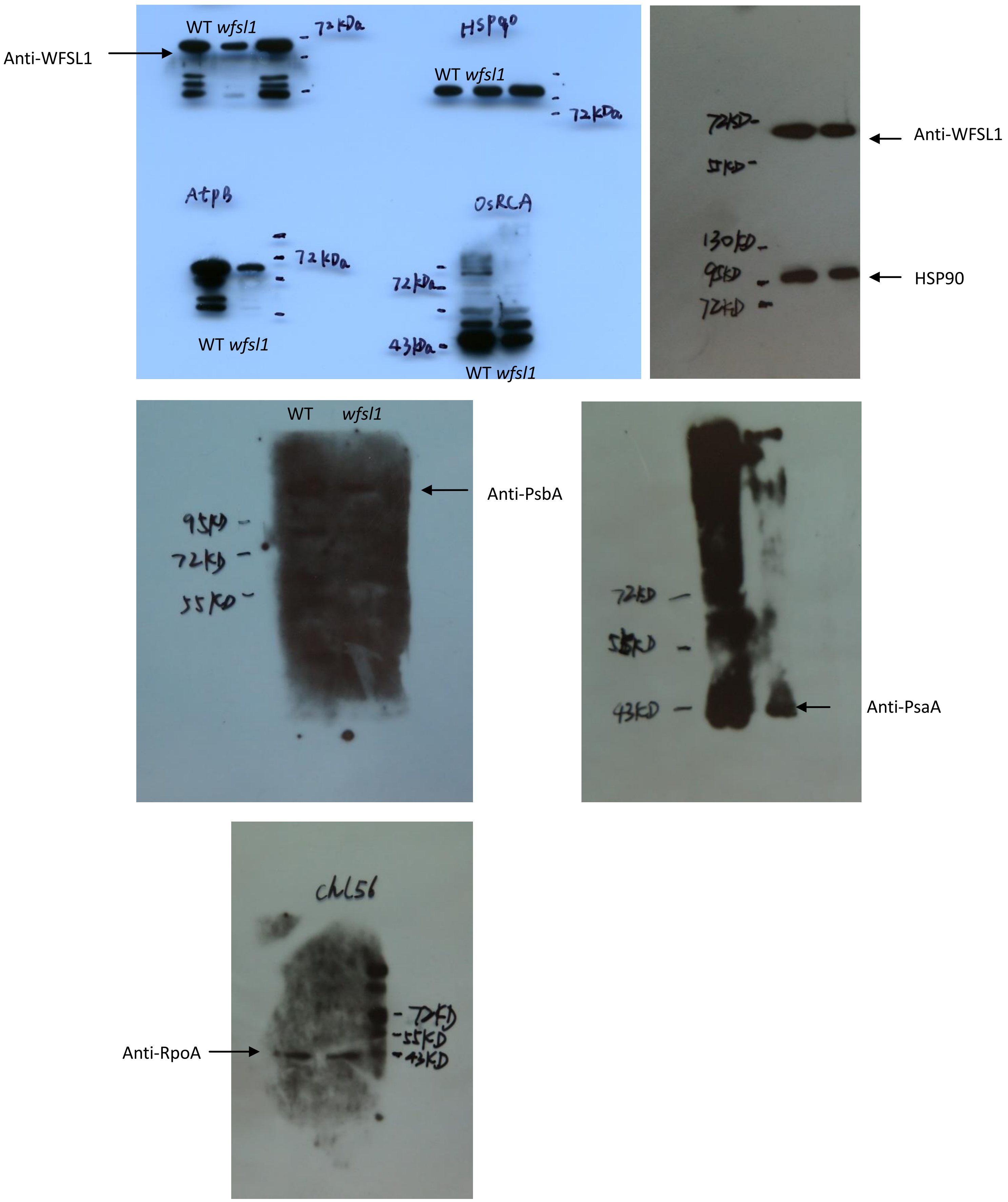

**Supplementary Figure S5 SDS-PAGE and RNA gel of wild type and *wfsl1***

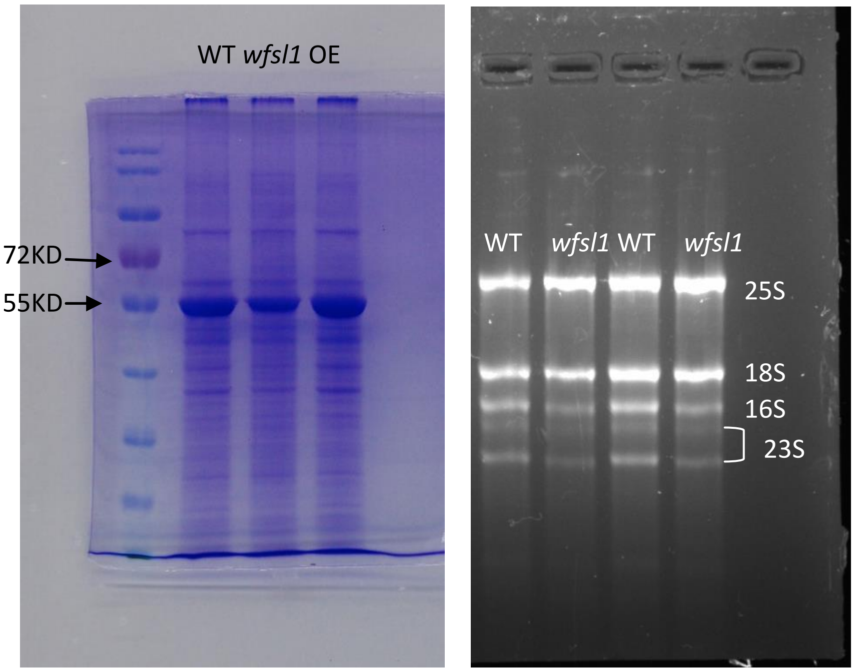

## Supplementary Tables

### Supplementary Table S1. Primers for cloning *WFSL1*

| Primers | Forward (5'-3') | Reverse (5'-3') |
|---|---|---|
| RM3252 | GGTAACTTTGTTCCCATGCC | GGTCAATCATGCATGCAAGC |
| gcw6 | TCGTTACCCAAGTTTTGAGAGC | TCAAACCATGTACCTTTCAGGA |
| gcw7 | CGCTCGCACCATTGATACTA | GATGCCAATCCCGAACTATC |
| gcw8 | CCCCCATCTCTTTAACAGTTTT | AAGGCTTTACCACTTCTGTTCC |
| gcw17 | GCCCTTCGACAAAAACAAAC | CTTGGAGCTTAGCTGGGGTA |
| gcw24 | CAAAGCTGTGGGATGTCAAA | TCTACCCAGGACGAACAAATC |
| gcw34 | CATTCCACTGTATTAGCCATCTG | ACACCACAGCAACGACTGAA |
| gcw35 | CAACAATGCCAAGGGAACAT | TGCCGTTTCTCCTCTGAACT |
| gcw56 | CCCAACCACTCATCATCCAT | GCGAAGATTGCTTCTTCCTT |
| RM5336 | TCGATTTGGTCGCGATTG | AGAAATCCCCGACCACCTC |

### Supplementary Table S2. Genetic analysis of *wfsl1*

| Cross | $F_1$ | | Number of wild type | Number of mutant type | $\chi^2$(3:1) | *P* value |
|---|---|---|---|---|---|---|
| | Wild type | Mutant | | | | |
| *wfsl1*/TN1 | 24 | 0 | 526 | 167 | 0.3006 | 0.5835 |
| *wfsl1*/93-11 | 10 | 0 | 245 | 70 | 1.2963 | 0.2549 |
| *wfsl1*/SH527 | 20 | 0 | 385 | 116 | 0.9108 | 0.3399 |

### Supplementary Table S3. Real-time PCR primers in this study

| Gene | Forward (5'-3') | Reverse (5'-3') |
|---|---|---|
| *WFSL1* | TCCACCAAGCAGGTCTTCG | CCTCTGAAACTCCTCCGTATC |
| *OsPORA* | ATGGCTCTCCAAGTTCAG | TGGCTCACGCTAAGGAAC |
| *OsPORB* | CCGCAAGGAGGGAGCGGTG | CCCTCTTGGTGCTAAGGCCG |
| *OsCAO1* | TTGGCTCAGTTAATGAGGGCAGAATCC | GGATGCGCACGTTGAGCATCTTTGTGG |
| *OsCAO2* | GATCCATACCCGATCGACAT | CGAGAGACATCCGGTAGAGC |
| *NOL* | CCACGAAAGGTATAGGATATG | TCAAGTCAGTCACCGCAGAT |
| *SGR* | ACGTCCACTGCCACATCTC | AACGCCTTCAGAACCACGG |

| | | |
|---|---|---|
| *V1* | CTCGTCTCGAACCCTCCTC | GACCGGTCGACCTTGGAG |
| *V2* | GCAGCAGATCCGTGATTACA | GCTGCTCCTTGAATGTCCAC |
| *OsDVR* | AGCCCAGGTTCATCAAGGT | TGATCACCCTCTCGAAGAACT |
| *OsChlH* | AACTGGATGAGCCAGAAGAGA | AAATGCAAAAGACTTGCGACT |
| *UBQ5* | ACCACTTCGACCGCCACTACT | ACGCCTAAGCCTGCTGGTT |
| *PsaA* | TTGAGTGGCATGTACTTCCAT | CTGGGTCCAATGTGAGTAGGATC |
| *PsbA* | ACATCGGATGGTTCGGTGTT | TACCATCAATATCTACTGGA |
| *AtpB* | ATGAATGTTATTGGTGAGCCA | CTGTTCTGTGGCTTGCTCAA |
| *RCA* | AAGATGACGATCGAAAAGC | AGTACTGCTCAGCCAGCTG |
| *RbcL* | AATGCGACTGCAGGTACA | GTCATGCATTACAATAGGAA |
| *RpoB* | AGATAATGTTTCCTACCCTG | ATTGTTGATAAAACTCCAAA |
| *RpoC1* | TCGCGAAACTCTGCTTGGGA | CACATTGATGTAATGAAAGT |
| *RpoC2* | GGAGATATTCATTTTCC | TTTTGATTCCTTCGAATC |
| *RpoA* | AATATTCTACAATAGCAGG | CCATAGAGATTACTTCTCAA |
| *16S -rRNA* | GAACGGGTGAGTAACGCGTA | GATTTCTCCTTTTGCTCCTC |
| *23S-rRNA* | TAAGCATAGATCCGGAGATTC | GTTGTCTCTTGCCTGCTCATG |
| *18S-rRNA* | ACAGTTCGGGCCAAATCCTG | CTAAGCATCATTGGCTTGGT |
| *25S-rRNA* | ACAACGACCAATCCTGAA | AGGCAGGCTTATACCATTAC |
| *PSBW* | AACTACTCCAAGGAGGC | GCCATCGCCAGTAGCGA |
| *PSB28* | ACAGGTTCATGCGATTCA | TCACTTCTTGACGAACTGGA |
| *RCABP89* | AGCTCAAGGTGAAGGAGCTC | GATGGCCTGGACGAAGAATC |
| *RPL18* | AAGACTCAGTGTTTTCCGCT | AACCAGAGTGCATGACTTTG |
| *NDHM* | ACTTCATCCGCAAACTGCTA | GTTTGCCCATGCTGAAATT |
| *CAB2R* | AGCTCAAGGTGAAGGAGATC | TTGCCGGTGACGATGGCCT |
| *CLA1* | TCAACTACCCCATCCACATG | TTGGAGACGTGGAAGATGAC |
| *PSAH* | GAGAAGAGCGTCTACTTCGA | AGCCGTACAGGTCCCACT |
| *OS07G0558400* | AGATCATCGGCACCCGGTT | AGGCCGAACACCTCGGTGTA |
| *PETC* | AAGGGTGACCCGACGTA | GTGCACACGGCGTTGATC |
| *OS05G0574600* | ACCTGTGGCAAAACCTCCA | AGACGAGCACCAGCAGCGT |
| *CIPK14* | GCACATAAGTACTTCCAGC | AAGTCCCGGTGATACACAC |
| *LAC24* | TCAAGCTGCTGTTGTGGA | TGCTGGCATAGCTGACTAA |
| *OS11G0127600* | TCCAGGCTTCAGATTCCATC | GCTAGGGTTGAGGAATTTC |
| *OS02G0306401* | ACGAGCATTTATCCAAGGT | TTACCATACACCTCATCAG |
| *KSL7* | TAACAACGTGCGCAATGGCA | AGATAGCTCATCTGAGGAA |
| *CSLH3* | ATCTCGATCTCATGCGGAGA | GTCGTCACGAACATGTCGA |
| *CM-LOX1* | ACTCCGGCCAACATCCAA | GTTTCCCTCGCGAACTCCT |
| *CIPK15* | GAGAGGTATGAGTTGGGGA | CCAGATTCCTTGCATAGTGC |
| *CBP3* | ATCATGAAGCTTGTTGGGAC | TAGCAAAGTTTCCCTTCGC |

**Supplementary Table S4. Down-regulated genes by RNA-seq analysis (part)**

| Gene_id | Gene name | $log_2$.Fold_change | Gene description |
|---|---|---|---|
| OS01G0600900 | *CAB2R* | -1.4385 | Chlorophyll a-b binding protein 2, chloroplastic |
| OS01G0773700 | *PSBW* | -1.7927 | Photosystem II reaction center W protein, chloroplastic |
| OS01G0938100 | *PSB28* | -2.013 | Photosystem II reaction center PSB28 protein, chloroplastic |
| OS03G0125000 | *RPL5* | -1.2667 | 50S ribosomal protein L5, chloroplastic |
| OS03G0169100 | *RPE* | -1.1392 | Ribulose-phosphate 3-epimerase, chloroplastic |
| OS03G0592500 | *RCABP89* | -2.0491 | Chlorophyll a-b binding protein, chloroplastic |
| OS03G0828100 | *RPL18* | -1.3513 | 50S ribosomal protein L18, chloroplastic |
| OS04G0539000 | *NDHM* | -1.3306 | NAD(P)H-quinone oxidoreductase subunit M, chloroplastic |
| OS04G0659100 | *GLN2* | -1.3257 | Glutamine synthetase, chloroplastic |
| OS04G0691600 | *RPS17* | -1.8763 | 30S ribosomal protein S17, chloroplastic |
| OS05G0408900 | *CLA1* | -1.8089 | 1-deoxy-D-xylulose-5-phosphate synthase 1, chloroplastic |
| OS05G0560000 | *PSAH* | -2.1419 | Photosystem I reaction center subunit VI, chloroplastic |
| OS06G0101600 | *PETE* | -2.3804 | Plastocyanin, chloroplastic |
| OS06G0472000 | *MSRB1* | -1.1315 | Peptide methionine sulfoxide reductase B1, chloroplastic |
| OS06G0647100 | | -1.4487 | 50S ribosomal protein L35 |
| OS07G0556200 | *PETC* | -1.4047 | Cytochrome b6-f complex iron-sulfur subunit, chloroplastic |
| OS08G0104600 | *ADI1* | -1.9831 | Ferredoxin-1, chloroplastic |
| OS09G0346500 | *CAB1R* | -1.1358 | Chlorophyll a-b binding protein 1, chloroplastic |
| OS11G0707000 | *RCA* | -1.0536 | Ribulose bisphosphate carboxylase/oxygenase activase, chloroplastic |
| OS12G0274700 | *RBCS* | -3.0721 | Ribulose bisphosphate carboxylase small chain, chloroplastic |
| OS12G0291100 | *RBCS-A* | -1.2709 | Ribulose bisphosphate carboxylase small chain A, chloroplastic |
| OS12G0420400 | | -1.2583 | Os12g0420400 protein; Photosystem I reaction center subunit XI, chloroplast |
| OS01G0749200 | | -1.0863 | Ribosomal protein L13 family protein |
| OS01G0805000 | | -1.3665 | Ribosomal protein L34 |
| OS03G0196800 | | -1.208 | Chloroplast 30S ribosomal protein S10 |
| OS01G0278900 | | -1.5925 | plastid-specific 50S ribosomal protein 5 |
| OS03G0122200 | | -1.0168 | 50S ribosomal protein L11, chloroplast |
| OS03G0284400 | | -1.1244 | Os03g0284400 protein; Ribosomal protein L10 containing protein |
| OS03G0769100 | | -1.1371 | 30S ribosomal protein S9, chloroplast |
| OS03G0815400 | | -1.51 | 50S ribosomal protein L17 |
| OS03G0843400 | | -1.1514 | 50S ribosomal protein L18, chloroplastic |
| OS03G0856500 | | -1.1587 | Os03g0843400 protein; Putative plastid ribosomal protein S6; Ribosomal protein S6 family protein |
| OS05G0101400 | | -1.0118 | Os03g0856500 protein; Plastid-specific 30S ribosomal protein 1, chloroplast |
| OS02G0189000 | | -1.0785 | Os02g0189000 protein; Ribosomal protein S21-like protein |
| OS02G0259600 | | -1.0957 | Os02g0259600 protein; Putative 50S ribosomal protein L21, chloroplast |

| | | |
|---|---|---|
| OS02G0652600 | -1.4384 | Os02g0652600 protein; Putative plastid ribosomal protein L19 |
| OS02G0754300 | -1.1889 | Os02g0754300 protein; Putative ribosomal protein L29 |
| OS03G0219900 | -1.0473 | 50S ribosomal protein L15, chloroplast |
| OS03G0284400 | -1.1244 | Os03g0284400 protein; Ribosomal protein L10 containing protein |
| OS03G0843400 | -1.1514 | Os03g0843400 protein; Putative plastid ribosomal protein S6 |
| OS03G0856500 | -1.1587 | Os03g0856500 protein; Plastid-specific 30S ribosomal protein 1, chloroplast |
